# Severity in the ICD-11 personality disorder model: Evaluation in a Spanish mixed sample

**DOI:** 10.3389/fpsyt.2022.1015489

**Published:** 2023-01-09

**Authors:** Fernando Gutiérrez, Anton Aluja, Claudia Rodríguez, Miguel Gárriz, Josep M. Peri, Salvador Gallart, Natalia Calvo, Marc Ferrer, Alfonso Gutiérrez-Zotes, Joaquim Soler, Juan Carlos Pascual

**Affiliations:** ^1^Institute of Neuroscience, Hospital Clínic of Barcelona, Barcelona, Spain; ^2^Institut d’Investigacións Biomèdiques August Pi i Sunyer (IDIBAPS), Barcelona, Spain; ^3^Lleida Institute for Biomedical Research Dr. Pifarré Foundation, Lleida, Spain; ^4^Department of Psychology, University of Lleida, Lleida, Spain; ^5^Neuropsychiatry and Drug Addiction Institute, Parc de Salut Mar, Barcelona, Spain; ^6^Department of Psychiatry, Mental Health, and Addiction, GSS–Hospital Santa Maria, Lleida, Spain; ^7^Department of Psychiatry, Vall d’Hebron University Hospital, Barcelona, Spain; ^8^Network Centre for Biomedical Research in Mental Health (CIBERSAM), Barcelona, Spain; ^9^Psychiatry and Legal Medicine Department, Autonomous University of Barcelona, Barcelona, Spain; ^10^Pere Virgili Health Research Institute (IISPV), CERCA, Reus, Spain; ^11^Pere Mata Psychiatric University Hospital, Reus, Spain; ^12^Hospital de la Santa Creu i Sant Pau, Barcelona, Spain

**Keywords:** personality disorder, personality pathology, severity, ICD-11, PDS-ICD-11

## Abstract

Severity is the main component of the ICD-11 personality disorder (PD) classification, but pertinent instruments have only recently been developed. We analyzed the psychometric properties of the *ICD-11 Personality Disorder Severity* scale (PDS-ICD-11) in a mixed sample of 726 community and clinical subjects. We also examined how the different components of the ICD-11 PD system —five trait domains, the borderline pattern specifier, and severity, all of them measured through self-reports— are interconnected and operate together. PDS-ICD-11 properties were adequate and similar to those of the original instrument. However, regressions and factor analyses showed a considerable overlap of severity with the five personality domains and the borderline specifier (72.6%). Bifactor modeling resulted in a general factor of PD (g-PD) that was not equivalent to severity nor improved criterion validity. The whole ICD-11 PD system, i.e., five personality domains, borderline, and severity, explained an average of 43.6% of variance of external measures of well-being, disability, and clinical problems, with severity contributing 4.8%. Suggestions to further improve the ICD-11 PD taxonomy include remodeling the present definition of severity to give more weight to the real-life consequences of traits.

## 1. Introduction

Severity is the most important component of personality disorder (PD) diagnosis in the newer *International Classification of Diseases, 11^th^ Revision* (ICD-11) (1). The diagnostic process first involves rating the subject into five levels of disturbance, from none to severe PD. The most prominent personality traits can then be optionally described according to five domains — negative affectivity, detachment, dissociality, disinhibition, and anankastia— plus a borderline pattern specifier based on the *Diagnostic and Statistical Manual of Mental Disorders*, 5th Edition (DSM-5) (2).

Whereas the relevance of PD severity —termed personality dysfunction in the DSM-5—, has been fostered for 30 years (3–5), its definition has been changing all the while (6). Some definitions have relied upon the consequences of traits, such as impairment in several life areas, subjective distress, risk of serious self-injury or death, risk to others, or service utilization (7, 8). Other definitions have revolved around symptomatic complexity, including the total number of PD criteria or disorders, the involvement of more than one cluster, or the presence of particular diagnoses, mostly borderline (3, 9, 10). In this case, severity is assumed to capture comorbidity between individual disorders, and is tightly related to the notion of a general factor that underlies all specific PDs (g-PD) (11–15). Still other definitions, rooted in the psychoanalytic literature, have focused on dysfunction of the self, such as compromised reality testing, primitive defense mechanisms, and identity diffusion (5, 16). For the time being, there is insufficient evidence on how these diverse conceptions of severity relate to each other or whether any of them should be preferred over the rest (7, 8). In fact, whereas the initial ICD-11 proposal accentuated major problems in occupational, social, and/or personal relationships (17), the final version incorporated self-dysfunction in order to bring itself into line with the DSM-5 (18–20), and both approaches are now pretty similar (21–23).

Among the instruments developed to measure severity, the *Standardized Assessment of Severity of Personality Disorder* (SASPD) (24) was a premature attempt, as it did not yet reflect the last-minute changes in the definition (21). An updated proposal are the still unnamed scales by Clark et al. (25) that, despite acceptable psychometric properties, may need additional improvements before clinical use, and are deemed preliminary by the authors. Finally, the *ICD-11 Personality Disorder Severity* scale (PDS-ICD-11) (26), which is the focus of the present study, reflects in a one-to-one basis the ICD-11 requirements for severity and has worked well in its original version. However, its properties have been tested on community subjects, except for limited analyses in 87 clinical subjects (26, 27).

It is clear from the above that many critical aspects of ICD-11 severity remain to be tested. The psychometric properties of the PDS-ICD-11 need to be replicated in clinical samples and in different cultures and languages. Among these properties, careful consideration should be given to dimensionality. Although severity is usually depicted as a unidimensional construct reflecting the dysfunctional features of any PD (18, 28–30), it actually encompasses very disparate or even antithetic features. For example, the PDS-ICD-11 conceives severity as an aggregate of either too low or too high sense of self-worth, insufficient or excessive goal orientation, imperious or no need for close relationships, too lax or too tight control over emotions, and so on. It is improbable that all these aspects and their opposites are equally relevant for maladaptation, so a more fine-grained analysis is warranted. Furthermore, it would be also unlikely for all personalities to show the same way of being dysfunctional. For example, fearful, psychopathic, or asocial personalities could be equally severe at the topmost level of abstraction, but may be related to quite different “types of severity” at lower levels (31). Thus, understanding severity will require careful examination at the item level.

Another underexplored aspect is the joint functioning of the whole ICD-11 PD system, that is, how its different components —personality domains, borderline specifier, and severity— relate to each other and operate together. Concerning the architecture of the diagnostic system, a twofold model that separates personality traits from severity/functioning has been gaining ground in current classifications (5, 8, 18, 32–35). This model, however, requires personality and severity to be two different things in the first place, which is currently far from clear. This separation could not be proved in the case of DSM-5 personality functioning, which massively overlaps with traits (23, 36–42), and this might be the case of the ICD-11 as well. Indeed, the scant evidence suggests that self-dysfunction is factorially inseparable of negative affectivity and disinhibition, and that interpersonal dysfunction is mixed with negative affectivity and detachment (25). Similar findings have been reported using diverse measures of personality and functioning (36, 43, 44), so the relationships between the descriptive and valuative components of the ICD-11 classification need further examination.

Finally, few data exist on the diagnostic utility of severity within the ICD-11 system. Previous operationalization of this construct have generally shown to be strong predictors of maladjustment, comorbid psychopathology, well-being, and treatment outcome (10, 13, 18, 22, 28, 45–47), and this could also be the case of the PDS-ICD-11 (26). The point is, however, whether the same information could be provided more parsimoniously by personality traits, either individually or in the form of a g-PD factor, making severity superfluous. To date, the contribution of severity seems modest at best, both in predicting categorial PDs (31, 38, 39, 41, 48–50) and external dysfunction criteria (51).

It is apparent from the above that further research is needed regarding some aspects of the ICD-11 classification of PDs. We still lack a sufficiently established measure of severity, as well as a clear idea of how the ICD-11 diagnostic system works as a whole. Our study aims 1) to analyze the psychometric properties of the Spanish version of the PDS-ICD-11 in a mixed community and clinical sample, with particular emphasis on dimensionality, 2) to examine how the different components of the ICD-11 PD system —the five trait domains, the borderline pattern specifier, and severity— interact with each other, and 3) to test how these components operate together as predictors of external measures of psychosocial impairment.

## 2. Materials and methods

### 2.1. Participants

The community sample consisted of 436 volunteers, 50.5% women, with mean age 46.3 years (SD = 18.0, range 18-87). Participants were undergraduates and their relatives and acquaintances recruited from a university in Spain. This sample was representative of the general Spanish population in terms of age (43.4 years)^1^ and level of education: about 17% had completed primary and lower secondary education, 37% upper secondary and post-secondary education, and the remaining 46% tertiary education. The clinical sample consisted of 290 outpatients, 67.9% women, with mean age 41.2 years (SD = 14.8, range 18–80). They were consecutively referred to the mental health units of six hospitals in Catalonia, Spain. Patients were clinically diagnosed at their respective centers, with the main diagnoses including mild to moderate affective disorders (25.2%), anxiety or phobic disorder (20.9%), mixed affective and anxious disorder (28.8%), eating disorder (1.8%), substance-related disorder (0.7%), and other disorders (22.6%) each with a frequency below 2%. No categorical diagnoses of personality disorder were made. With α = 0.05 and 1-β = 0.80, the combined sample allowed detecting correlations of 0.11. The study was approved by the ethical committees of the respective centers.

### 2.2. Instruments and procedure

Questionnaires were delivered to community participants by undergraduates taking part in a personality research and practice program. Participants answered anonymously and did not receive any compensation for participating. Clinical subjects filled the questionnaires in their respective hospitals, as a part of their diagnostic procedure. All questionnaires were completed individually, in paper-and-pencil format, and in the same order they are presented below.

The *Personality Inventory for ICD-11* (PiCD) (52) is a 60-item Likert-type self-report measuring the five personality domains of the dimensional ICD-11 personality model: negative affectivity, detachment, dissociality, disinhibition, and anankastia (1). Each domain has 12 items rated from 1 (strongly disagree) to 5 (strongly agree). With a similar format, the *Borderline Pattern Scale* (BPS) (53) includes 12 items and was developed to accommodate the DSM-5 borderline diagnosis (2) into the ICD-11 classification. Both instruments have shown good psychometric properties in their Spanish versions (54, 55).

The *ICD-11 Personality Disorder Severity Scale* (PDS-ICD-11) (26) is a 14-item measure designed to assess the various components of PD severity in the ICD-11: four items are related to self-dysfunction (identity, self-worth, self-perception, and goals); four to interpersonal dysfunction (interest in relationships, perspective-taking, mutuality, and disagreement management); five to control over emotions, behavior, and cognition; and one measures global psychosocial impairment. The instrument was translated by four Spanish native speakers who were familiar with the constructs being measured and worked independently. The translators agreed on a common version, which was blindly back-translated by an English native speaker and compared with the original. Discrepancies were consensually resolved by the translators and the authors of the instrument.

The *Level of Personality Functioning Scale–Brief Form 2.0* (LPFS-BF 2.0) (56) is a 12-item self-report measuring the self-functioning and interpersonal-functioning components of personality dysfunction described in Section III of the DSM-5. It has shown good reliability and validity in different samples and languages (57).

Subjective well-being was measured through the *World Health Organization-5 Well-Being Index* (WHO-5), a 5-item self-report that has been widely used to measure the impact of mental problems on quality of life (58). It measures levels of mood, energy, and interest for things in a 0-100 scale, with higher scores indicating greater well-being. Disability was measured through the 12-item self-reported *World Health Organization Disability Assessment Schedule 2.0* (WHODAS 2.0) (59), which assesses difficulties due to health conditions in six different life areas: understanding and communication, self-care, mobility, interpersonal relationships, work and household roles, and community and civic roles. Scores range from 0 (no disability) to 48 (total disability). The *Work and Social Adjustment Scale* (WSAS) (60) is a 5-item self-reported measure of general impairment assessing the impact of an identified problem, mental condition in this case, on five areas: work, home management, social leisure, private leisure, and relationships, each rated on a scale of 0 to 8. Higher scores in a 0-40 range denote more disability.

## 3. Data analysis and results

### 3.1. PDS-ICD-11 psychometric properties

All analyses were made using R package ‘psych’ (61) and SPSS v. 25 (62) unless stated otherwise. Descriptive statistics for the ICD-11 components and the remaining instruments are shown in Supplementary Table 1. The PDS-ICD-11 showed good internal consistency, with Cronbach’s alpha of α = 0.89 and McDonald’s omega of ω = 0.93 based on the one-factor solution. All corrected item-scale correlations were r_*i–s*_ > 0.50 except for item 13 (r_i–s_ = 0.44).

An item response theory (IRT) analysis based on Samejima’s graded response model was undertaken using the “ltm” package (63) to analyze the information provided by each item (Supplementary Table 2). Items 9 (emotional control) and 2 (self-worth) showed the highest discrimination ability with a > 2.50, whereas item 13 (harm to others) showed the lowest (0.95). Items 11 (experience of reality), 12 (harm to self), and particularly item 13 (harm to others, b_3_ = 5.15), showed high difficulty in parameter b_3_, questioning the utility of the most extreme response option (“*I often harm others”*) (Supplementary Figure 1). This item also offered the least information (Supplementary Figure 2). The test information function shows that the highest reliability came about at theta values between 0 and 2 (Supplementary Figure 3).

The unidimensionality of the PDS-ICD-11 was tested through confirmatory factor analysis (CFA) in R package “lavaan” (64), using weighted least squares estimation. Results suggested adequate fit, with χ2 = 193.81, df = 77, *p* < 0.001, Comparative Fit Index CFI = 0.993, Tucker-Lewis Index TLI = 0.992, Root Mean Square Error of Approximation RMSEA = 0.046 and Standardized Root Mean Square Residual SRMR = 0.051 (Supplementary Table 2). All loadings were above 0.60 except for items 11 (experience of reality) and 13 (harm to others). Factor structure was invariant between the community and clinical samples, as given by the “sirt” package (65).

### 3.2. Structure of the whole ICD-11 system: PiCD, BPS, and PDS-ICD-11

#### 3.2.1. Correlation and regression analyses

**FIGURE 1:**
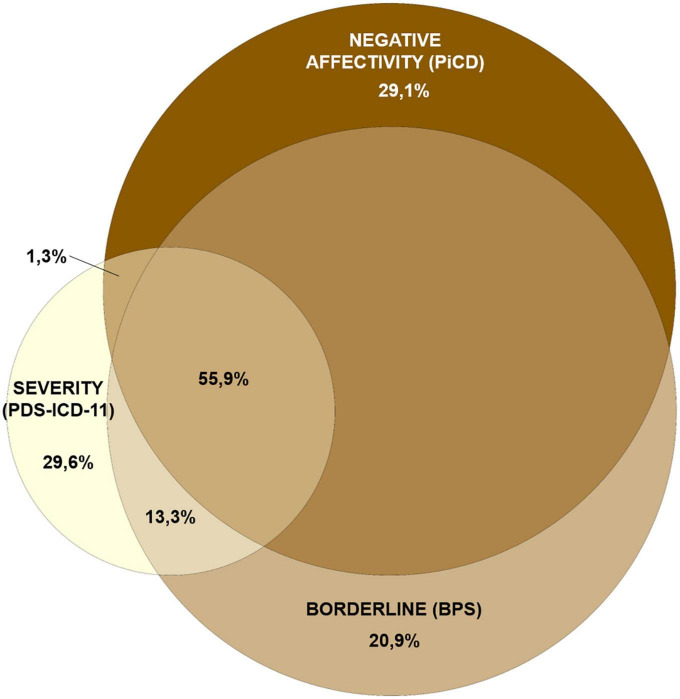
Euler diagram showing the overlap between negative affectivity, borderline, and severity. Circle sizes are proportional to variances. Due to the geometric impossibility to represent the overlap between more than three variables through a proportionally-built Euler diagram, anankastia and detachment —explaining an additional 2% of the PDS-ICD-11 variance— have been omitted. Drawn at http://eulerr.co/.

Pearson’s and disattenuated intercorrelations between the five personality domains, the borderline specifier, and severity are shown in Table 1. Strong associations were found of severity with borderline (0.83), negative affectivity (0.76), and PiCD total score (0.72), which rose to 0.92, 0.84, and 0.80 after disattenuation for reliability (66). The considerable overlap between severity, negative affectivity, and borderline can be better appreciated through an Euler diagram (Figure 1). Associations were moderate with the remaining personality domains and negligible with anankastia. Accordingly, multiple regression analyses showed that 72.6% of the variance of severity is explained by the five PiCD domains and the borderline specifier. Borderline was the best predictor of severity (beta = 0.527), followed by negative affectivity (Table 1, below). Disinhibition and dissociality showed little or no relationship with severity even after the borderline specifier was excluded, and anankastia showed an inverse association.

**TABLE 1:** Pearson’s (lower triangle) and disattenuated correlations (upper triangle) between the ICD-11 system components with alphas in the diagonal, and multiple regressions of severity on personality traits.

	PiCD negative affect	PiCD detachment	PiCD disinhibition	PiCD dissociality	PiCD anankastia	BPS total	PDS-ICD-11
**Correlations**							
PiCD negative affect	0.90	0.44	0.63	0.42	0.08	0.91	0.84
PiCD detachment	0.38	0.85	0.41	0.36	0.19	0.53	0.54
PiCD disinhibition	0.55	0.34	0.83	0.66	-0.54	0.72	0.65
PiCD dissociality	0.37	0.31	0.56	0.86	-0.21	0.55	0.50
PiCD anankastia	0.07	0.16	-0.44	-0.17	0.79	-0.14	-0.16
BPS total	0.84	0.47	0.64	0.49	-0.12	0.92	0.92
PDS-ICD-11	0.76	0.47	0.56	0.44	-0.13	0.83	0.89
	**Standardized betas**						**R^2^**
**Regressions**							
Five domains + BPS	**0.290**	**0.147**	-0.055	0.029	-**0**.**138**	**0**.**527**	0.726
Five domains	**0.660**	**0.232**	-0.021	**0**.**093**	-**0**.**213**	−	0.664

PiCD, personality inventory for ICD-11; BPS, borderline pattern scale; PDS-ICD-11, ICD-11 personality disorder severity scale. Correlation coefficients > 0.10 are significant at *p* < 0.01. Disattenuated correlations are Pearson’s correlations divided by the square root of the product of the reliabilities of each pair of variables ($r_{c}=\frac{r_{x\,y}}{\sqrt{\omega_{x}\times \omega_{y}}}$). Significant regression coefficients are in boldtype.

#### 3.2.2. Item-level analysis

Additionally, we wanted to clarify whether certain personality traits were associated to specific aspects of severity. To this end, two different scoring procedures for the PDS-ICD-11 were used: Whereas in the standard unipolar scoring the two poles of each item are added together and contribute interchangeably to a single dimension of severity (2-1-0-1-2), we adopted an alternative bipolar procedure in which the two poles pointed in opposite directions (0-1-2-3-4). Under unipolar scoring, Spearman’s correlations showed that all PDS-ICD-11 items were homogeneously correlated with the borderline specifier (mean r_s_ = 0.50), negative affectivity (0.47) and PiCD total score (0.47), although with some advantage for self-worth and psychosocial impairment (Supplementary Table 3). By contrast, associations were more specific under bipolar scoring (Supplementary Table 3, below): negative affectivity and borderline became more associated to low self-worth and emotional dysregulation, whereas detachment was weakly but differentially linked to low interest in relationships (r_s_ = 0.27), anankastia to rigid goals and behavioral overcontrol (0.25 and 0.41), disinhibition to difficulty following goals and lack of behavioral control (0.34 and 0.41), and dissociality to more disagreements and lack of behavioral control (0.28 and 0.27). However, each aspect of severity is measured by a single item in the PDS-ICD-11, and associations were generally not strong.

#### 3.2.3. Factor analysis

The structure of the whole ICD-11 system, operationalized by the PiCD, the BPS, and the PDS-ICD-11, was then examined at the item-level through exploratory factor analysis based on the polychoric matrix (Supplementary Table 4) and unweighted least squares estimation. Kaiser-Meyer-Olkin index was 0.874 and Bartlett’s sphericity test was 7,569.7 (df = 3,655; *p* = 0.00001), indicating suitability of data for factor analysis. Velicer’s MAP and optimal implementation of parallel test (67) suggested four and five factors, respectively. One to seven factors were successively retained, rotated to Promin, and examined. The four-factor solution (Supplementary Table 5) reproduced the usual structure containing disinhibition-anankastia (*r* = −0.79 and 0.85 with the original domain scores), dissociality (0.95), and detachment (0.94), along with a broader factor gathering negative affectivity, borderline, and severity together (0.94, 0.94, and 0.87). Solutions of five or more factors produced empty or trivial factors, and were unsuccessful in separating this latter factor into its original components.

Additionally, we adopted a pure exploratory bifactor approach (68) implemented in FACTOR 12.01.02 (69) to clarify whether a general factor of personality disorder (g-PD) could be extracted from the ICD-11 personality descriptors —five domains and the borderline specifier— and whether this g-PD would be equivalent to severity. KMO was 0.902 and Bartlett’s statistic 7,842.2 (df = 2,556; *P* = 0.00001). MAP and parallel test suggested retaining four and five specific factors, respectively. Whereas the former solution had one empty and one uninterpretable factor, the five-factor solution showed a clear g-PD with 72% of items loading over 0.30 and five specific factors roughly representing detachment (*r* = 0.56 with the original domain), negative affectivity-borderline (0.67 and 0.62), dissociality (0.64), anankastia (0.56), and a factor gathering disinhibition items but with little relation with the original domain (0.29) (Supplementary Table 6). The g-PD was mostly associated to negative affectivity and borderline (0.70 and 0.73) and moderately to the remaining personality domains (0.29 to 0.62), but could not be regarded as exactly equivalent to severity (0.66) or the LPFS-BF (0.69).

### 3.3. Concurrent and criterion validity of the ICD-11 system

#### 3.3.1. Correlation and regression analyses

We examined the relationships of the ICD-11 components with external indicators of maladaptation: caseness (belonging to the clinical sample, as an indicator of clinically significant problems), as well as three World Health Organization (WHO) scales reflecting well-being and disability. Severity showed the strongest correlation with all indicators (mean r = 0.60), albeit with little advantage over negative affectivity (0.59), borderline (0.57), and the DSM-5 LPFS-BF (0.58), with which severity correlated 0.81. PiCD total and the g-PD showed slightly lower averages (0.52 and 0.49), whereas anankastia showed no relation to maladaptation (Table 2). Due to the extensive overlap among ICD-11 components, the predictive utility of diverse combinations of these components was then analyzed through multiple regression. The whole ICD-11 system —five personality domains (PiCD), the borderline specifier (BPS), and severity (PDS-ICD-11)— predicted 40.6% of the variance of the WHODAS, 45.8% of the WHO-5, 47.9% of the WSAS, and 39.9% of caseness (Table 2, below). The five personality domains alone were better predictors than severity alone. However, both severity and the personality domains added incremental variance to each other (4.8 and 6.3%, respectively). Among personality domains, disinhibition, dissociality, and anankastia were non-significantly or inversely related to maladaptation. The exclusion of the borderline specifier was inconsequential for prediction. Interactions of personality traits with severity, which reflect the additional contribution of having an extreme trait *plus* high severity, were non-significant too.

**TABLE 2:** Correlations of the ICD-11 components with external indicators of well-being, disability, and clinical problems, and multiple regressions of indicators on ICD-11 components.

	WHO-5	WHODAS	WSAS	Caseness
**Correlations**				
PiCD negative affect	-0.60**	0.56**	0.62**	0.59**
PiCD detachment	-0.40**	0.41**	0.33**	0.33**
PiCD disinhibition	-0.36**	0.38**	0.38**	0.33**
PiCD dissociality	-0.18**	0.20**	0.23**	0.21**
PiCD anankastia	0.07	0.02	-0.03	0.00
PiCD total	-0.51**	0.53**	0.53**	0.50**
g-PD	-0.49**	0.49**	0.52**	0.47**
BPS total	-0.59**	0.54**	0.60**	0.55**
PDS-ICD-11	-0.61**	0.58**	0.66**	0.59**
DSM-5 LPFS-BF	-0.60**	0.57**	0.60**	0.53**
**Regressions (% exp. variance)**				
5 domains + PDS-ICD-11 items	42.9%	48.3%	50.0%	43.4%
All ICD-11 components	40.6%	45.8%	47.9%	39.9%
5 domains + severity	40.6%	45.7%	47.9%	39.9%
5 domains alone	36.2%	42.2%	40.1%	36.2%
Severity alone	33.8%	37.4%	43.8%	33.8%

PiCD, personality inventory for ICD-11; g-PD, general factor of personality disorder; BPS, borderline pattern scale; PDS-ICD-11, ICD-11 personality disorder severity scale; LPFS-BF, level of personality functioning scale-brief form; WHO-5, World Health Organization-5 well-being index; WHODAS 2.0, World Health Organization Disability Assessment Schedule 2.0; WSAS, work and social adjustment scale. Caseness is dichotomous, so point-biserial correlations are shown, but linear regressions were used for the sake of comparison (125, 126). Alternative logistic regressions gave Cox & Snell’s pseudo-R^2^ coefficients 2% smaller. **p < 0.01.

#### 3.3.2. Item-level analysis

However, the best predictions resulted from replacing the PDS-ICD-11 scale with its items in regression analyses. All models invariably retained items 2 and 14, suggesting that self-worth and global psychosocial impairment are particularly relevant aspects of severity. Other items made smaller contributions that fluctuated across models. Mean incremental variance of items over the five personality domains was 7.4% (Table 2). Supplementary Figure 4 shows that most PDS-ICD-11 items are more maladaptive in one of their extremes. Namely, having a weak sense of identity, feeling worthless, perceiving few strengths in oneself, lacking emotional control, losing touch with reality under stress, harming oneself or others, and showing psychosocial impairment in several important areas of life (items 1 to 3, 9, and 11 to 14), are more detrimental than their opposites. By contrast, having too rigid goals and being over-controlled are just as bad as lacking any goal at all and being impulsive (items 4 and 10). Similarly, in the interpersonal domain, being uninterested in relationships, unempathetic, selfish, or adversarial (items 5 to 8) is almost as bad as being afraid of loneliness and disagreements, or being too kind or clingy.

#### 3.3.3. ROC analysis

**FIGURE 2:**
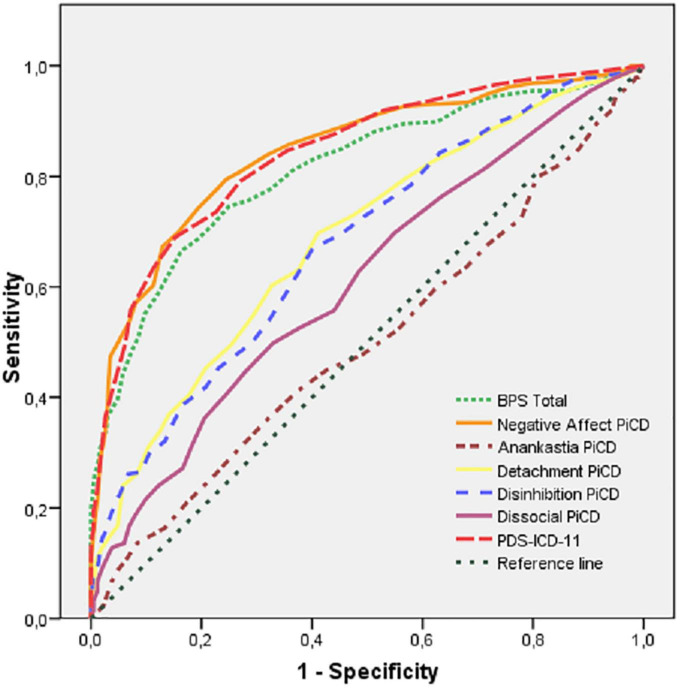
ROC curves for the components of the ICD-11 PD system predicting caseness.

Finally, the seven components of the ICD-11 PD system were tested as for their ability to predict caseness through receiver operating characteristics (ROC) analysis. Negative affectivity and severity obtained virtually identical areas under the curve (AUC 0.844 and 0.842, respectively), closely followed by borderline (0.812) (Figure 2). The remaining scales showed AUCs between 0.501 and 0.684. Although the Youden’s index suggested a cutpoint of 10 (resulting in 69% sensitivity and 85% specificity), a still lower threshold of 8 would be necessary to detect at least 80% of cases whereas still discarding 73% of non-cases. The proposed cutoff of 17.5 (26, 27) would only detect about 22% of cases.

## 4. Discussion

The PDS-ICD-11 shows good psychometric properties in its Spanish version, similar to those of the original and the German versions (26, 27). Specifically, it has high internal consistency and a well-fitted unidimensional structure, and it is among the best predictors of external indicators of well-being, disability, and clinical problems. This makes it a suitable instrument for measuring the severity dimension of the ICD-11 PD system.

On the other hand, some unexpected results suggest that there remains scope for improvement. The ICD-11 diagnostic system does not explicitly assume that personality domains and severity are independent constructs. Rather, elevations in severity are expected to be associated to elevations in one or several personality domains, which reflect distinct styles of malfunctioning. However, what is unanticipated is the ample overlap between personality traits and severity/dysfunction which has been repeatedly found regarding both the ICD-11 and DSM-5 systems (25, 36, 38, 70). In our study, almost three quarters of the variance of severity is accounted for by personality descriptors, particularly the negative affectivity domain and the borderline specifier. In fact, these three components cannot be split and are virtually the same thing from a factor-analytic perspective (25, 55). A major consequence is that severity plays a smaller role than expected in predicting maladaptation, and borderline plays no role whatsoever; for this purpose, negative affectivity alone would work almost as well. This result is rather expected for the borderline specifier, which was appended to the system at the eleventh hour and shows little specific variance (55), but it deserves further consideration regarding severity. As a second point, we could not confirm either that severity reflects the maladaptation resulting from a wide range of dysfunctional personality styles. Instead, regression analyses reveal that the PDS-ICD-11 provides little or no coverage of the possible impairments linked to disinhibition, dissociality, and anankastia (Table 1). This has been also the case with the German PDS-ICD-11 regarding the two latter domains (27).

One possible explanation is that, indeed, certain traits are harmful whereas others are innocuous. For example, negative emotionality has proven to be definitely impairing with regard to a broad range of clinical outcomes, whereas all other dimensions are associated to only a few detrimental outcomes or are clinically inconsequential (71). In fact, anankastic features such as perfectionism, workaholism, rigidity, or even the obsessive-compulsive PD, have shown to be fairly harmless (29, 72, 73), or even beneficial in certain areas (71, 74), and the same is true of disinhibition and dissociality (71, 75–77). In accordance with this, these domains are also unrelated in our study to either caseness or the WHO scales. A second possibility is that the ICD-11 and DSM-5 unidimensional constructs of severity/dysfunction are pervaded by the frequency and intensity of aversive emotions, which is the defining feature of negative affectivity, but they do not reflect the type of impairment characteristic of most other traits. Only when we take advantage of the bipolar nature of the PDS-ICD-11 items (Supplementary Table 3) we can appreciate that anankastia causes subjects to adhere rigidly to unreachable goals and impulse control, that detachment results in social isolation, that dissociality turns disagreements into major conflicts, and that disinhibition prevents subjects from attaining their predefined goals. Thus, the unidimensional construct of severity/dysfunction may overdetect distress-related problems but be blind to the many other ways of being maladapted. A third possibility is that, whatever it is the domain that is causing problems in the first place, most patients may ultimately seek help because of distress and demoralization, which would thrust both severity and negative affectivity at the same time (78). This is consistent with the fact that all domains (except anankastia) are significantly associated to severity until negative affectivity is controlled for through regression (Table 1). A final possibility is that it is the PDS-ICD-11, not the severity construct in itself, which is biased toward negative affect. In this case, clinician ratings, interviews, or other self-reports available in the near future could be better able to differentiate severity from negative affect, or to capture the maladaptive aspects of disinhibition, dissociality, and anankastia. For example, it has been reported that personality functioning overlaps with traits to a lesser extent when it is measured longitudinally through daily diaries (79). By contrast, the only study using clinician ratings of ICD-11 severity shows results which are very similar to those of self-reports (80). All these possible explanations are not mutually exclusive.

No consensual solution to these shortcomings has been found so far, and the debate is ongoing (28). Given that severity is the pivotal component of the system, and that personality domains are only optional ways to identify particular styles of malfunctioning, it has been proposed that it is domains which should be amended or replaced with normal-range traits to reduce overlap (6, 42, 81). Normal traits, however, such as those of the Five Factor Model, have shown to be equally related to severity, so the problem is left unsolved (6, 39). A more statistically sophisticated solution is the extraction of a general factor of PD (g-PD) that accounts for the common variance across disorders or traits (11, 12, 15). This approach significantly reduces the overlap between domains (82), and the resulting g-PD is a good indicator of maladaptation, thus precluding the need for a separate assessment of severity (13, 14, 30). However, the g-PD does not work well in our study: It is not equivalent to either ICD-11 severity or DSM-5 personality functioning, and it does not bring significant advantage over preexisting variables in predicting well-being and disability. An additional drawback of this approach is that it provides the clinician with a broad factor whose meaning we can barely envisage (83–85), and a number of specific factors with no less ambiguous significance. For example, one might wonder what negative emotionality would look like after distress and impairment have been removed, if such a thing exists at all, and what is the point of having a so inert domain in a diagnostic system.

Thus, psychometric refinement may not be sufficient to break this deadlock, mainly caused by the fact that severity and negative affectivity are largely the same. Another proposed solution has been the elimination of severity in its current form (38, 40). However, severity is upheld not only on empirical but also on theoretical grounds, and so its conceptual foundations need previous clarification and discussion. Impairments in self- and interpersonal functioning have been emphasized in many widely accepted models of PD, as those of Kernberg, Livesley, Parker, or Cloninger (16, 86–88). A central assumption underpinning these models and, by extension, the ICD-11 and DSM-5 systems, is that the main components of severity/dysfunction —self-direction, sense of identity, empathy, intimacy— are of a different nature than personality traits. Concretely, they have been considered to be core features, basic psychological capacities, meta-constructs, key components of an intrapsychic system needed to fulfill universal life tasks, or even the cornerstone of humanness (5, 28, 86, 88–91). These appellations do not clarify, however, what exactly makes them different from all remaining traits (92). For example, there is no reason to think that these features reflect capacities or serve universal life tasks to a greater extent than any other trait. In fact, all traits reflect the variation of brain systems that enable and impel us to perform essential life-sustaining tasks: detecting and managing threats, exploring the surroundings, striving for incentives, gaining power or status, becoming attached to others, deterring rivals, and dozens of equally —if no more— important pursuits (93–97). We also lack evidence to assert that identity or empathy are either more universal or consequential, or are more central to PDs, than all others. And certainly, we do not know which —if any— is the cornerstone of humanness. In essence, self- and interpersonal dysfunctions may simply reflect the fact that most PDs are underlain by neuroticism and disagreeableness (98, 99). A second, closely related assumption of current classifications is that the components of severity/dysfunction are inherently maladaptive. For example, whereas negative affectivity, disinhibition, or dissociality need an additional criterion to be considered pathological in the ICD-11 system, the deleterious nature of low empathy or self-directedness is taken for granted. In practice, this leads to a tautological diagnostic process in which the severity of some traits is determined on the basis of other —or even the same— traits. On empirical grounds, this is at odds with the well-known fact that negative affectivity is the most maladaptive trait ever found (71, 100, 101). By contrast, the premise that self-complexity, low self-directedness, aloofness, or lack of empathy are dysfunctional in themselves has no comparable support (71, 75–77, 102–104). Although the abovementioned assumptions are difficult to prove or refute at this time, they certainly warrant in-depth examination and debate with a view to future revisions of the taxonomy.

A promising alternative may be the replacement —or the complementation— of the current severity construct with an assessment of the negative consequences of traits (33, 71, 92, 105–108). Negative consequences have been categorized in many ways, but they generally include difficulties or failures in a number of key areas: *education/work*, e.g., inability to finish studies, hold a paid job, or achieve financial independence; *interpersonal functioning*, e.g., trouble finding or maintaining romantic relationships, chronic conflict with family, or lack of a support network; *social functioning*, e.g., difficulties for life in society, breaking rules of coexistence, or harming others; *physical health and longevity*, e.g., problems for self-care, harming oneself, or putting oneself at risk of death; and *psychological health*, e.g., chronic suffering, substance abuse, psychopathology, requiring specialized care or hospitalization, or being unable to attain acceptable levels of well-being (71, 109–111). This is a redress rather than a turning point, as the PDS-ICD-11 already encompasses outcomes such as harm to self, harm to others, or psychosocial impairment. However, the focus is moved further from theory-driven intrapsychic constructs towards concrete outcomes in the real world, which may bring a number of interwoven advantages. First, life outcomes are clearly different from traits, which reduces conceptual confusion and redundancy. Second, this approach recovers a more pragmatic view of PDs as extreme traits causing suffering or functional impairment (112), rather than as pathological entities characterized by poorly known intrapsychic processes. Third, it puts the accent on functional status, the most difficult aspect to attain and maintain in severe PD patients, and then a primary goal for intervention (113). Fourth, it gives us the chance of exploring from scratch what traits change what aspects of our lives for the worse (or for the better). As a final advantage, it excludes the possibility of diagnosing a PD in the absence of negative consequences in the real world (33), and thus the risk of pathologizing normal behavior. In doing so, it offsets the increasing trend toward assuming that PDs are defects, illnesses, or dysfunctions *sensu* Wakefield: the failure of a mental mechanism to perform its evolved function (34). On the contrary, a life-outcome perspective focuses on whether traits are harmful —negative or undesirable by social standards (34, 114–117)— and then fits in better with the fact that, at present, no evidence supports a dysfunction model for PDs. Whereas we know what normal lungs and hearts look like and what they are expected to do, this is not the case of “normal” personality (118). On the other hand, what is normal in nature, from insects to higher primates and humans, is the coexistence of different personalities, often maintained by balancing selection (119, 120). For example, certain traits may damage important biological goals while promoting others, or may be globally beneficial or detrimental depending on environmental circumstances, thus remaining in the population at evolutionary equilibrium. The very existence of a single “normal” personality would then be an evolutionary anomaly (121, 122).

Whereas this approach may lay the foundations for a less conjectural and more pragmatic taxonomy of difficult personalities, some caution is needed. On the one hand, it is not as objective as it may seem, as we cannot establish which life outcomes are undesirable and which constitute a ‘good life’ without a significant amount of subjectivity and theorizing (114). On the other, personality functioning and psychosocial impairment are different constructs. Whereas the former is about what do all PDs have in common (e.g., low self-directedness, impaired mentalizing, the g-PD), and then about the mechanisms of personality pathology (28, 123), the latter refers to the impact of traits on the life of individuals. It is argued, therefore, that the consequences of disease are essential in clinical decision making but are not the disease itself, and cannot be part of diagnosis (28). In turn, this assumes that PDs are diseases rather than disliked traits, a point on which we are far from a consensus (114–117). Ultimately, even if both approaches are deemed complementary, life outcomes have been relatively overlooked in current classifications and deserve greater consideration they have received so far.

Some caveats on the scope of this study are in order. First, the instruments are not the model, so that the shortcomings identified in the ICD-11 diagnostic system could be circumscribed to specific questionnaires such as the PiCD, the BPS or the PDS-ICD-11. Moreover, all instruments are self-reported, which may produce common-method bias and overestimate overlapping (124). Therefore, the generalizability or our results depends on their eventual confirmation using different tools, preferably interviews or clinician rating forms, when they are developed. Second, the same is true of criterion variables. Particularly, caseness cannot be considered equivalent to PD diagnosis, but it is a general indicator of clinically significant problems. Other criteria suggestive of disordered personality need to be tested in upcoming studies. Third, certain parts or our analysis are performed at the item level. Even if severity is a complex construct and its components need to be examined separately, it should be taken into account that items are less reliable than scales, and these results should be interpreted cautiously. Finally, our sample included less than 300 patients. This is enough to reach our study’s aims but did not allow deeper examination of different levels of severity, which is an important feature of the ICD-11 system.

With these objections in mind, we conclude that the PDS-ICD-11 has proven adequate properties as a measure of severity. However, the ICD-11 system as a whole is conditioned by important limiting factors. Whereas the adoption of a dimensional taxonomy has meant a significant improvement, the overlap between personality domains —one major reason for the abandonment of traditional categories (35)— remains a problem (81, 82). Furthermore, while the conceptual separation between personality traits and severity/dysfunction is widely accepted and helpful, the existing operationalizations of severity lack solid theoretical justification and are too close to negative affectivity to serve diagnostic purposes. Irrespective of whether this is due to personality traits containing variance of impairment (29) or the other way around (38, 39), the result is a redundant and conceptually confusing diagnostic system. In contrast, the types of impairment resulting from disinhibition, dissociality, and anankastia are not well reflected by severity, or these traits are not significantly harmful, as suggested by previous research (71, 74). We take up previous suggestions that a model with refined, truly independent domains (82), followed by a list of undesired life consequences would be more parsimonious, feasible, and theoretically clearer than the current approach (33, 106).

## Data availability statement

The datasets presented in this study can be found in online repositories. The names of the repository/repositories and accession number(s) can be found below: https://osf.io/mvyug/?view_only=94d52cde2dd540b09665d775a9eea52e.

## Ethics statement

The studies involving human participants were reviewed and approved by Comité de Ética de Investigación con Medicamentos (CEIm, Drug Research Ethics Committee), Hospital Clínic, Barcelona, Spain. The patients/participants provided their written informed consent to participate in this study.

## Author contributions

FG, AA, CR, MG, and JMP contributed to the conception and design of the study. FG and CR performed the statistical analyses and wrote the first draft of the manuscript. CR, SG, NC, MF, AGZ, JS, and JCP made amendments to the manuscript and rewrote parts of it. All authors recruited samples in their respective centers, assessed outpatients, organized the database, contributed to the manuscript revision, and approved the submitted version.

## Footnotes

1
www.ine.es
