# Supplementary material for: Severity in the ICD-11 personality disorder model: Evaluation in a Spanish mixed sample
Source: Front Psychiatry. 2023 Jan 9;13:1015489. doi: 10.3389/fpsyt.2022.1015489 (PMC9868964; doi:10.3389/fpsyt.2022.1015489)
Supplement: Supplementary file 1 [file Data_Sheet_1.PDF]

Supplementary Material

Severity in the ICD-11 Personality Disorder Model: Evaluation in a Spanish Mixed Sample

Supplementary Table S1. Descriptive Statistics and Mean Differences Between the Community and Clinical Samples for the Study Variables.

	Total				Community		Clinical		
	ω	M (SD)	Skew	Kurt	ω	M (SD)	ω	M (SD)	p
PDS-ICD-11	.93	8.3 (6.2)	.839	.317	.88	5.4 (4.0)	.91	12.8 (6.2)	<.001
PiCD – Negative Affect.	.92	34.3 (10.4)	.198	-.745	.87	29.4 (7.8)	.90	41.8 (9.3)	<.001
PiCD – Detachment	.88	26.9 (8.5)	.583	.094	.87	24.6 (7.3)	.87	30.3 (9.0)	<.001
PiCD – Disinhibition	.89	24.9 (8.4)	.696	.025	.88	22.7 (7.1)	.89	28.3 (9.1)	<.001
PiCD – Dissocial	.89	22.0 (7.3)	1.054	1.043	.88	20.7 (6.4)	.90	23.8 (8.1)	<.001
PiCD – Anankastia	.82	39.1 (7.1)	-.321	.159	.81	39.1 (6.8)	.83	39.1 (7.6)	.916
BPS	.95	26.1 (11.2)	.853	.080	.90	21.1 (7.2)	.95	33.7 (11.8)	<.001
LPFS-BF	.94	23.2 (7.9)	.625	-.078	.91	20.0 (5.8)	.93	28.6 (8.1)	<.001
WHO-5	.92	13.7 (5.3)	-.425	-.516	.89	15.9 (4.0)	.91	10.1 (5.3)	<.001
WHODAS 2.0	.95	20.9 (9.2)	1.169	.806	.93	17.2 (6.3)	.92	27.1 (9.9)	<.001
WSAS	.93	9.9 (10.6)	.910	-.271	.90	4.5 (6.3)	.86	18.8 (10.2)	<.001

Note. ω = McDonald's omega reliability; M = mean; SD = standard deviation; Skew = skewness; Kurt = Kurtosis; PDS-ICD-11 = ICD-11 Personality Disorder Severity Scale; PiCD = Personality Inventory for ICD-11; BPS = Borderline Pattern Scale; LPFS-BF = Level of Personality Functioning Scale-Brief Form; WHO-5 = World Health Organization-5 Well-Being Index; WHODAS 2.0 = World Health Organization Disability Assessment Schedule 2.0; WSAS = Work and Social Adjustment Scale.

Supplementary Table S2. Factor Loadings from the CFA Model, Item Discrimination and Item Difficulty Parameters for the PDS-ICD-11.

		IRT Parameters			
	Factor Loadings	a	b_1	b_2	b_3
1. Identity	.69	1.68	.60	2.24	-
2. Self-worth	.82	2.54	.26	1.76	-
3. Self-perception	.73	1.95	1.06	2.69	-
4. Goals	.68	1.62	-.29	2.01	-
5. Interest in relationships	.73	1.85	.56	2.19	-
6. Perspective taking	.64	1.44	.14	1.81	-
7. Mutuality in relationships	.80	2.30	.44	1.98	-
8. Disagreement management	.68	1.62	-.15	1.90	-
9. Emotional control	.83	2.56	.06	1.61	-
10. Behavioral control	.76	2.06	.16	1.96	-
11. Experience of reality	.59	1.27	-.67	1.23	3.54
12. Harm to self	.66	1.47	.19	1.26	2.94
13. Harm to others	.50	.95	-.63	2.19	5.15
14. Psychosocial impairment	.78	2.20	-.96	.62	1.54

Note. PDS-ICD-11 = ICD-11 Personality Disorder Severity Scale.

Supplementary Figure S1. Item Characteristic Curves of the PDS-ICD-11 Items.

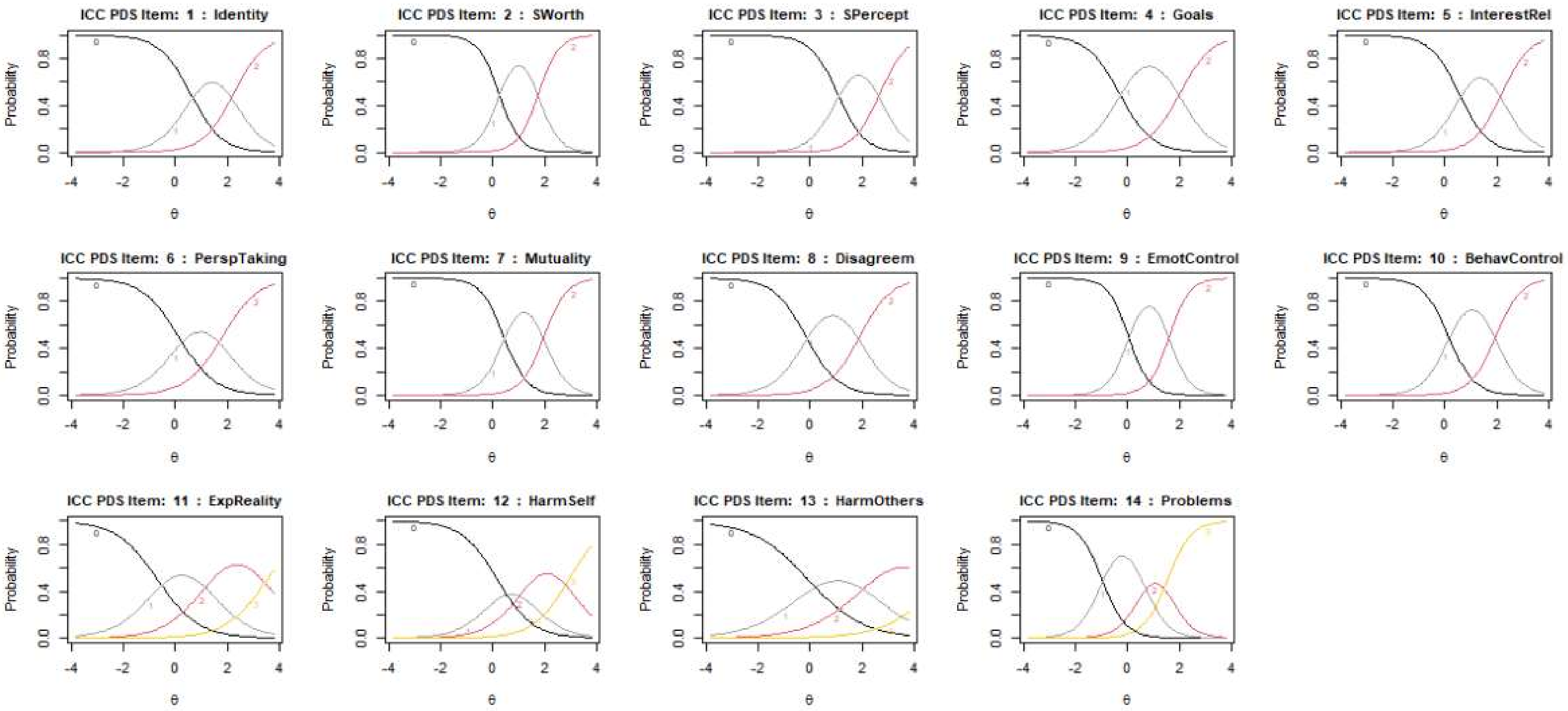

Supplementary Figure S2. Item Information Curves of the PDS-ICD-11 Items.

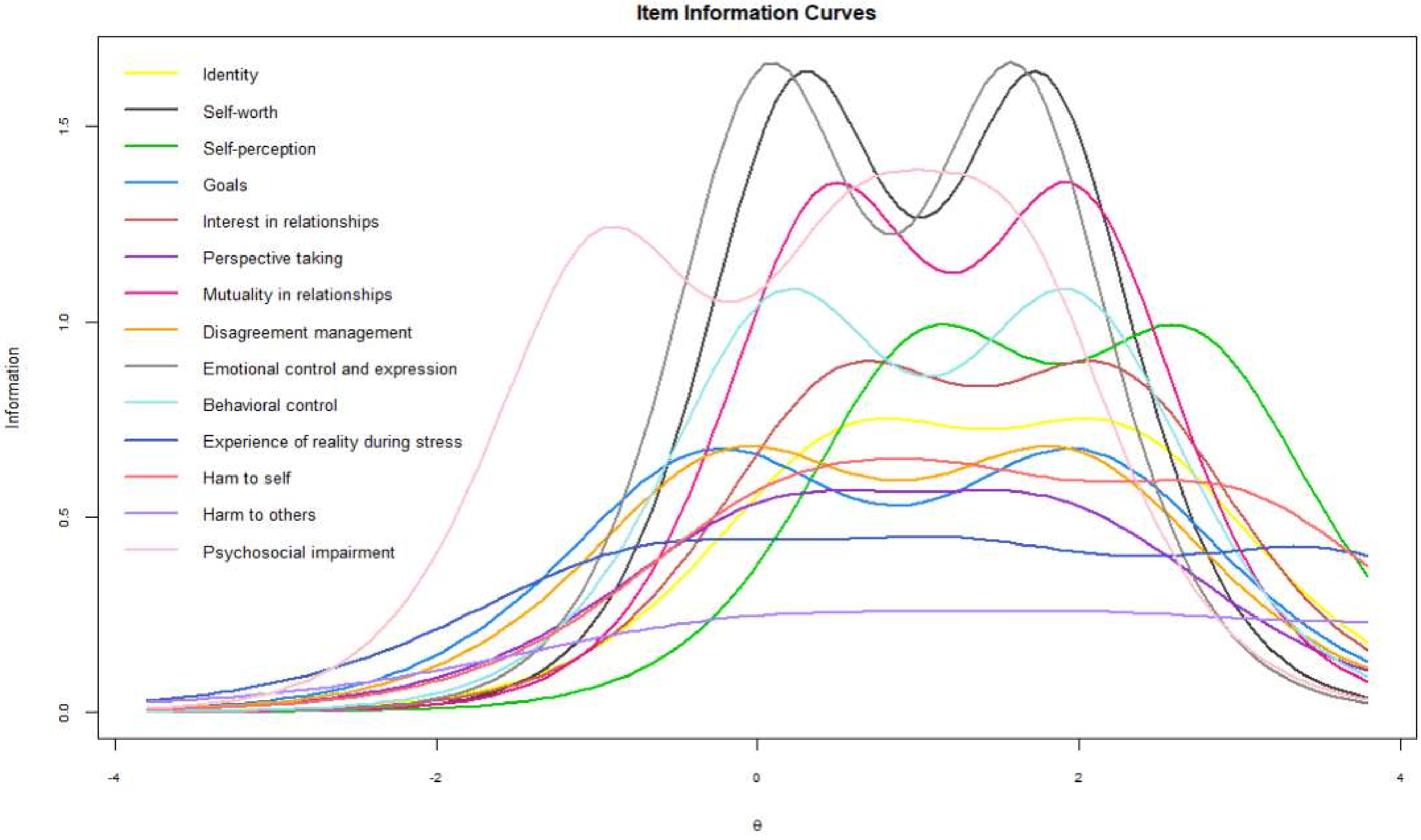

Supplementary Figure S3. Test Information Function of the PDS-ICD-11.

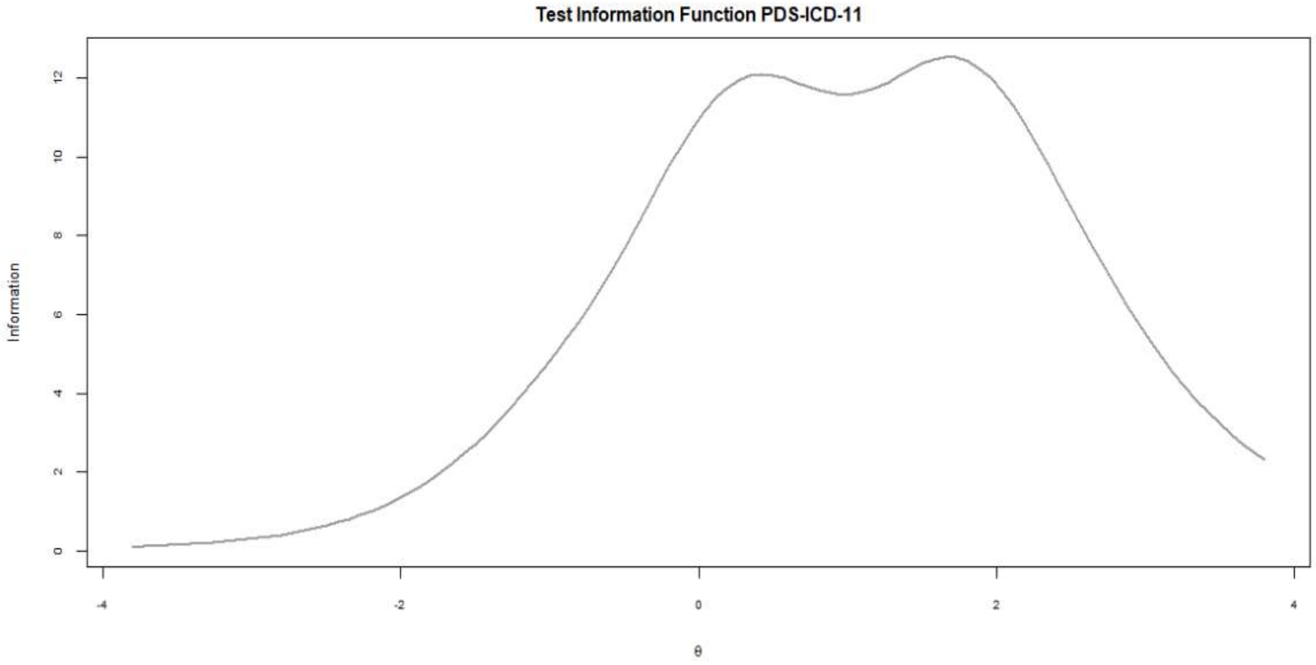

Supplementary Table S3. Spearman's Correlations of the PDS-ICD-11 Items with the Five Personality Domains and the Borderline Qualifier Under Unipolar and Bipolar Scoring.

	PiCD Negative affect.	PiCD Detach-ment	PiCD Disinhi-bition	PiCD Disso-ciality	PiCD Anan-kastia	PiCD Total	BPS Total
Unipolar scoring							
1. Identity	.46**	.31**	.32**	.25**	-.02	.48**	.48**
2. Self-worth	.62**	.40**	.37**	.25**	.01	.59**	.62**
3. Self-perception	.38**	.29**	.25**	.10**	-.09*	.36**	.40**
4. Goals	.48**	.23**	.34**	.17**	-.08*	.41**	.48**
5. Interest relationships	.44**	.31**	.35**	.21**	-.05	.45**	.49**
6. Perspective taking	.43**	.25**	.28**	.24**	.00	.43**	.44**
7. Mutuality	.51**	.33**	.41**	.27**	-.05	.53**	.52**
8. Disagr. management	.43**	.27**	.30**	.20**	-.06	.41**	.46**
9. Emotional control	.52**	.36**	.41**	.28**	-.13**	.53**	.59**
10. Behavioral control	.53**	.33**	.39**	.27**	-.08*	.52**	.56**
11. Experience of reality	.51**	.20**	.30**	.21**	-.02	.42**	.52**
12. Self-harm	.43**	.28**	.30**	.28**	-.14**	.41**	.50**
13. Others-harm	.28**	.16**	.29**	.38**	-.18**	.31**	.35**
14. Psychosocial impairment	.61**	.32**	.39**	.30**	-.05	.56**	.65**
Bipolar scoring							
1. Identity	-.24**	-.14**	-.17**	-.09*	.11**	-.20**	-.27**
2. Self-worth	-.60**	-.33**	-.30**	-.09*	-.01	-.50**	-.57**
3. Self-perception	-.38**	-.25**	-.25**	-.03	.12**	-.31**	-.40**
4. Goals	-.26**	-.20**	-.34**	-.06	.25**	-.23**	-.29**
5. Interest relationships	.02	-.27**	.02	-.05	-.03	-.11**	-.01
6. Perspective taking	.35**	.02	.11**	-.01	.08*	.22**	.28**
7. Mutuality	.31**	.14**	.14**	.01	.09*	.25**	.29**
8. Disagr. Management	-.06	-.01	-.19**	-.28**	.19**	-.13**	-.12**
9. Emotional control	-.42**	.04	-.25**	-.16**	.11**	-.26**	-.39**
10. Behavioral control	-.16**	.03	-.41**	-.27**	.41**	-.15**	-.22**

Note. PDS-ICD-11 = ICD-11 Personality Disorder Severity Scale; PiCD = Personality Inventory for ICD-11; BPS = Borderline Pattern Scale; ** p < .01, * p < .05; Items 11 to 14 are unipolar and then identical across both scoring methods.

Supplementary Table S4. Polychoric Correlations Matrix of the PDS-ICD-11 Items.

PDS-ICD-11 items	1. Identity	2. Self-worth	3. Self-perception	4. Goals	5. Interest relationships	6. Perspective taking	7. Mutuality	8. Disagr. management	9. Emotional control	10. Behavioral control	11. Experience of reality	12. Self-harm	13. Others-harm	14. Psychosocial impair.
1. Identity	—													
2. Self-worth	.60	—												
3. Self-perception	.59	.63	—											
4. Goals	.49	.57	.57	—										
5. Interest relationships	.49	.54	.53	.52	—									
6. Perspective taking	.46	.55	.46	.44	.49	—								
7. Mutuality	.52	.65	.54	.58	.57	.57	—							
8. Disagr. management	.37	.52	.52	.44	.47	.48	.57	—						
9. Emotional control	.51	.62	.59	.47	.62	.51	.66	.59	—					
10. Behavioral control	.49	.58	.50	.43	.54	.43	.57	.51	.69	—				
11. Experience of reality	.46	.46	.40	.36	.43	.39	.45	.43	.53	.51	—			
12. Self-harm	.44	.51	.44	.35	.43	.35	.42	.37	.53	.44	.45	—		
13. Others-harm	.27	.27	.21	.27	.23	.16	.36	.34	.39	.35	.30	.56	—	
14. Psychosocial impair.	.54	.70	.49	.53	.57	.43	.59	.49	.60	.61	.51	.58	.46	—

Note. PDS-ICD-11 = ICD-11 Personality Disorder Severity Scale.

Supplementary Table S5. Promin-Rotated Four-Factor Pattern for the ICD-11 System Components at the Item-Level.

	F1	F2	F3	F4
PiCD 35 Anankastia	**.776**	**.403**	.250	-.070
PiCD 5 Anankastia	**.745**	**.329**	.274	-.109
PiCD 55 Anankastia	**.681**	-.145	.060	.205
PiCD 15 Anankastia	**.674**	.021	-.104	.239
PiCD 20 Anankastia	**.671**	**.460**	**.327**	-.046
PiCD 45 Anankastia	**.661**	.057	.217	.206
PiCD 60 Anankastia	**.583**	-.049	.002	.116
PiCD 7 Disinhibition	**-.563**	.057	.078	.145
PiCD 12 Disinhibition	**-.532**	-.004	-.010	.221
PiCD 25 Anankastia	**.526**	-.037	.019	.266
PiCD 50 Anankastia	**.479**	.025	.259	-.181
PiCD 27 Disinhibition	**-.473**	.063	.072	.119
PiCD 47 Disinhibition	**-.449**	.163	**.379**	-.104
PiCD 57 Disinhibition	**-.432**	.070	.043	.216
PiCD 32 Disinhibition	**-.415**	.224	.208	-.096
PiCD 40 Anankastia	**.363**	.013	.181	.193
PiCD 42 Disinhibition	**-.332**	.198	-.048	.186
PiCD 59 Dissociality	-.013	**.824**	-.074	-.037
PiCD 44 Dissociality	.050	**.762**	.078	-.086
PiCD 54 Dissociality	-.104	**.750**	**-.330**	.184
PiCD 49 Dissociality	.004	**.729**	.062	-.124
PiCD 14 Dissociality	.232	**.728**	-.079	-.203
PiCD 19 Dissociality	.220	**.681**	-.074	-.019
PiCD 34 Dissociality	-.047	**.673**	-.004	.010
PiCD 9 Dissociality	-.034	**.656**	-.259	.112
PiCD 24 Dissociality	-.003	**.652**	**-.407**	.125
PiCD 39 Dissociality	-.125	**.634**	-.201	.196
PiCD 4 Dissociality	-.163	**.463**	**.313**	-.094
BPS 3 Borderline	-.100	**.330**	-.076	.265
PiCD 52 Disinhibition	-.289	**.319**	.179	.023
PiCD 22 Disinhibition	-.122	**.300**	.026	.224
BPS 1 Borderline	-.024	-.063	**.898**	.014
BPS 5 Borderline	-.014	.008	**.858**	.030
PiCD 16 Negative affect.	.197	.051	**.831**	-.015
PiCD 41 Negative affect.	.018	-.059	**.809**	.128
PiCD 1 Negative affect.	.073	.038	**.806**	-.068
PiCD 56 Negative affect.	.089	.144	**.803**	-.017
PiCD 11 Negative affect.	-.024	.008	**.785**	.018
PiCD 36 Negative affect.	.264	-.284	**.765**	-.176
PiCD 21 Negative affect.	.137	-.037	**.760**	.112
PiCD 46 Negative affect.	.081	-.143	**.750**	.163
BPS 2 Borderline	-.015	-.204	**.744**	.283
BPS 12 Borderline	-.089	.051	**.735**	.068
BPS 11 Borderline	.002	-.116	**.712**	.044
PDS 14 Psychosocial impair.	-.073	-.066	**.710**	.106
PDS 2 Self-worth	-.008	-.121	**.698**	**.304**
BPS 10 Borderline	-.042	-.020	**.698**	.210
PiCD 51 Negative affect.	.040	.030	**.694**	.194
PiCD 6 Negative affect.	**.378**	.203	**.646**	-.053
PDS 10 Behavioral control	-.100	.008	**.629**	.128

PiCD 31 Negative affect.	-.013	-.019	**.629**	-.039
PDS 7 Mutuality	-.084	-.058	**.628**	.185
PDS 11 Experience of reality	.013	-.026	**.603**	.038
PDS 9 Emotional control	-.201	-.052	**.601**	.199
BPS 6 Borderline	-.100	.171	**.594**	.120
BPS 4 Borderline	-.028	.158	**.587**	.054
PiCD 23 Detachment	.118	.202	**-.570**	**.317**
PDS 5 Interest relationships	-.102	-.094	**.565**	.215
PDS 4 Goals	-.172	-.208	**.564**	.184
PiCD 2 Disinhibition	-.221	.232	**.563**	-.186
BPS 8 Borderline	-.293	.069	**.562**	.113
PDS 8 Disagr. management	-.109	-.122	**.537**	.157
BPS 9 Borderline	.005	.244	**.531**	.049
PDS 6 Perspective taking	.027	-.007	**.529**	.114
BPS 7 Borderline	-.201	.181	**.527**	.192
PDS 1 Identity	-.046	-.018	**.522**	.253
PDS 12 Self-harm	-.156	-.004	**.485**	.133
PDS 3 Self-perception	-.169	-.124	**.477**	**.306**
PiCD 17 Disinhibition	**-.310**	.264	**.439**	-.126
PiCD 29 Dissociality	-.073	.265	**.306**	-.017
PDS 13 Others-harm	-.147	.252	**.300**	-.090
PiCD 43 Detachment	.130	-.104	.053	**.769**
PiCD 13 Detachment	.149	-.164	.161	**.758**
PiCD 33 Detachment	.078	.223	-.024	**.710**
PiCD 28 Detachment	.121	-.246	.094	**.690**
PiCD 58 Detachment	.088	.144	.257	**.615**
PiCD 3 Detachment	.127	.123	.281	**.567**
PiCD 48 Detachment	.017	.264	-.149	**.456**
PiCD 38 Detachment	.010	.133	.140	**.543**
PiCD 8 Detachment	-.029	.284	-.192	**.507**
PiCD 53 Detachment	-.066	**.411**	**-.370**	**.469**
PiCD 18 Detachment	-.022	**.329**	.103	**.426**
PiCD 30 Anankastia	**.365**	-.022	**-.332**	**.382**
PiCD 10 Anankastia	.247	-.124	-.001	.279
PiCD 26 Negative affect.	-.035	.195	.076	.202
PiCD 37 Disinhibition	-.245	.259	.119	.141
Correlations with domains				
PiCD Negative affect.	-.22**	.47**	**.94****	.39**
PiCD Detachment	-.12**	.38**	.38**	**.94****
PiCD Disinhibition	**-.79****	.67**	.62**	.35**
PiCD Dissociality	-.43**	**.95****	.44**	.24**
PiCD Anankastia	**.85****	-.18**	-.06	.13**
PiCD Total	-.27**	.73**	.80**	.67**
BPS Total	-.45**	.60**	**.94****	.50**
PDS-ICD-11	-.44**	.51**	**.87****	.53**

Note. PDS-ICD-11 = ICD-11 Personality Disorder Severity Scale; PiCD = Personality Inventory for ICD-11; BPS = Borderline Pattern Scale;. Relevant coefficients are in boldtype.

Supplementary Table S6. Promin-Rotated 5+1 Bifactor Pattern for the ICD-11 System Components at the Item-Level.

	GF	F1	F2	F3	F4	F5
PiCD 13 Detachment	**.439**	**.677**	.094	-.260	-.232	-.119
PiCD 43 Detachment	**.445**	**.663**	-.007	-.226	-.204	-.143
PiCD 33 Detachment	**.560**	**.592**	-.060	-.088	.091	-.037
PiCD 28 Detachment	**.355**	**.559**	.022	-.184	**-.339**	-.146
PiCD 8 Detachment	**.324**	**.492**	-.152	.012	.241	.003
PiCD 58 Detachment	**.654**	**.463**	.154	-.107	.024	-.061
PiCD 3 Detachment	**.558**	**.457**	.171	-.122	.041	.027
PiCD 53 Detachment	**.352**	**.407**	-.298	.128	.286	-.015
PiCD 48 Detachment	**.357**	**.386**	-.131	-.031	.167	-.037
PiCD 38 Detachment	**.569**	**.371**	.058	-.105	.060	-.132
PiCD 18 Detachment	**.535**	**.344**	.095	-.044	.267	-.030
BPS 1 Borderline	**.627**	-.167	**.685**	-.059	-.058	-.126
BPS 5 Borderline	**.664**	-.145	**.654**	-.104	-.013	-.141
PiCD 2 Disinhibition	**.310**	-.073	**.621**	.293	**.373**	.085
PiCD 11 Negative affect.	**.593**	-.141	**.608**	-.018	-.005	-.088
BPS 12 Borderline	**.601**	-.037	**.605**	-.040	.078	-.141
PiCD 41 Negative affect.	**.647**	-.074	**.600**	-.065	-.095	-.114
BPS 2 Borderline	**.577**	.132	**.589**	-.123	-.178	-.184
PiCD 56 Negative affect.	**.634**	-.202	**.588**	-.092	.067	.021
PiCD 16 Negative affect.	**.511**	-.163	**.588**	-.164	-.006	.133
PiCD 1 Negative affect.	**.497**	-.206	**.586**	.011	.023	.101
PiCD 17 Disinhibition	.297	.032	**.572**	**.327**	**.431**	.006
BPS 10 Borderline	**.598**	.099	**.570**	-.153	.011	-.173
BPS 8 Borderline	**.572**	.066	**.560**	.019	.177	**-.324**
PiCD 46 Negative affect.	**.541**	-.015	**.549**	-.108	-.172	-.076
PiCD 21 Negative affect.	**.535**	-.053	**.530**	-.133	-.077	.035
PiCD 47 Disinhibition	.299	.035	**.542**	**.369**	**.367**	-.179
PiCD 30 Anankastia	.110	.111	**-.526**	-.197	**-.329**	.084
PiCD 31 Negative affect.	**.355**	-.078	**.516**	.015	.008	.004
BPS 11 Borderline	**.478**	-.111	**.515**	-.028	-.100	-.064
BPS 7 Borderline	**.632**	.122	**.508**	-.079	.230	-.270
BPS 6 Borderline	**.628**	.006	**.499**	-.061	.161	-.178
PiCD 51 Negative affect.	**.631**	.007	**.492**	-.075	-.012	-.065
PiCD 23 Detachment	-.114	**.335**	**-.491**	.060	.091	.197
PiCD 36 Negative affect.	.208	**-.333**	**.476**	-.054	**-.372**	.163
BPS 4 Borderline	**.552**	-.048	**.458**	-.092	.123	-.105
PiCD 15 Anankastia	.085	-.100	**-.456**	-.273	**-.367**	**.436**
BPS 9 Borderline	**.560**	-.081	**.397**	-.027	.184	-.011
PiCD 27 Disinhibition	**.409**	.035	.111	**.674**	.111	-.254
PiCD 12 Disinhibition	**.409**	.140	.051	**.629**	.079	**-.370**
PiCD 7 Disinhibition	**.382**	.146	.208	**.418**	.212	**-.429**
PiCD 57 Disinhibition	**.414**	.155	.115	**.365**	.139	**-.357**
PiCD 42 Disinhibition	**.426**	.084	-.010	**.322**	.182	-.253
PiCD 59 Dissociality	**.487**	-.195	-.123	.096	**.639**	.165
PiCD 54 Dissociality	**.394**	.110	-.292	.086	**.636**	.045

PiCD 44 Dissociality	**.496**	-.240	-.007	.009	**.574**	.172
PiCD 49 Dissociality	**.440**	-.242	-.001	.076	**.566**	.175
PiCD 34 Dissociality	**.430**	-.030	.004	.061	**.557**	.095
PiCD 39 Dissociality	**.443**	.139	-.146	-.008	**.547**	-.074
PiCD 24 Dissociality	.210	.128	**-.302**	.011	**.506**	.088
PiCD 9 Dissociality	**.330**	.089	-.203	.036	**.505**	.059
PiCD 19 Dissociality	.285	-.118	-.163	.061	**.473**	**.408**
PiCD 4 Dissociality	**.446**	-.098	**.306**	.078	**.471**	-.027
PiCD 14 Dissociality	.284	**-.391**	-.219	.067	**.448**	**.388**
PiCD 32 Disinhibition	.196	.079	**.411**	**.343**	**.418**	-.135
PiCD 52 Disinhibition	**.423**	.015	.247	.059	**.362**	-.262
BPS 3 Borderline	.294	**.309**	.005	-.022	**.335**	-.060
PiCD 35 Anankastia	.116	-.242	-.093	-.244	.057	**.806**
PiCD 5 Anankastia	.037	-.215	-.021	**-.353**	.035	**.729**
PiCD 20 Anankastia	.246	-.195	.022	**-.305**	.147	**.654**
PiCD 50 Anankastia	-.027	**-.364**	-.039	.064	-.185	**.591**
PiCD 6 Negative affect.	**.421**	-.259	**.333**	-.027	.021	**.439**
PiCD 60 Anankastia	-.005	-.128	-.286	-.099	**-.364**	**.479**
PiCD 55 Anankastia	-.021	-.019	-.248	-.268	**-.453**	**.471**
PiCD 45 Anankastia	**.312**	-.185	-.194	-.269	**-.337**	**.431**
PiCD 25 Anankastia	.192	-.069	**-.319**	-.040	**-.373**	**.384**
PiCD 40 Anankastia	.284	-.087	-.101	.097	-.240	**.335**
PiCD 10 Anankastia	.128	.091	-.175	-.022	-.289	.120
PiCD 22 Disinhibition	**.366**	.203	.081	.083	.276	-.073
PiCD 26 Negative affect.	**.307**	.169	.097	.046	.139	-.039
PiCD 29 Dissociality	**.384**	-.095	.249	.128	.221	.036
PiCD 37 Disinhibition	**.437**	.098	.163	.072	.252	-.247
Correlations with domains						
PiCD Negative affect.	**.70****	-.18**	**.67****	-.07	-.16**	.10**
PiCD Detachment	**.62****	**.56****	-.20**	-.39**	.11**	-.41**
PiCD Disinhibition	.39**	.02	.39**	.29**	.30**	-.14**
PiCD Dissociality	**.52****	.14**	.00	-.14**	**.64****	-.22**
PiCD Anankastia	.29**	-.32**	-.32**	-.05	**-.51****	**.56****
PiCD Total	**.83****	.07	.24**	-.11**	.11**	-.05
BPS Total	**.73****	.06	**.62****	-.20**	.07	-.20**
PDS-ICD-11	**.66****	.12**	**.51****	-.21**	.08*	-.23**

Note. PDS-ICD-11 = ICD-11 Personality Disorder Severity Scale; PiCD = Personality Inventory for International Classification of Diseases, 11th Revision; BPS = Borderline Pattern Scale. Relevant coefficients are in boldtype.

Supplementary Figure S4. Level of impairment (%) for the 14 items of the PDS-ICD-11.

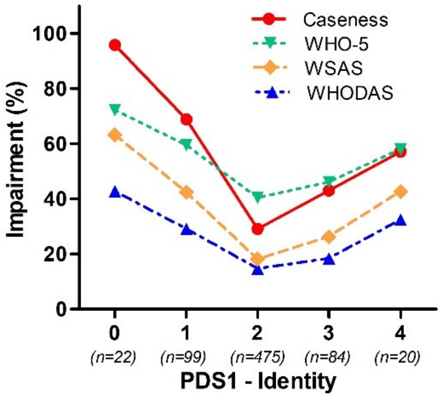

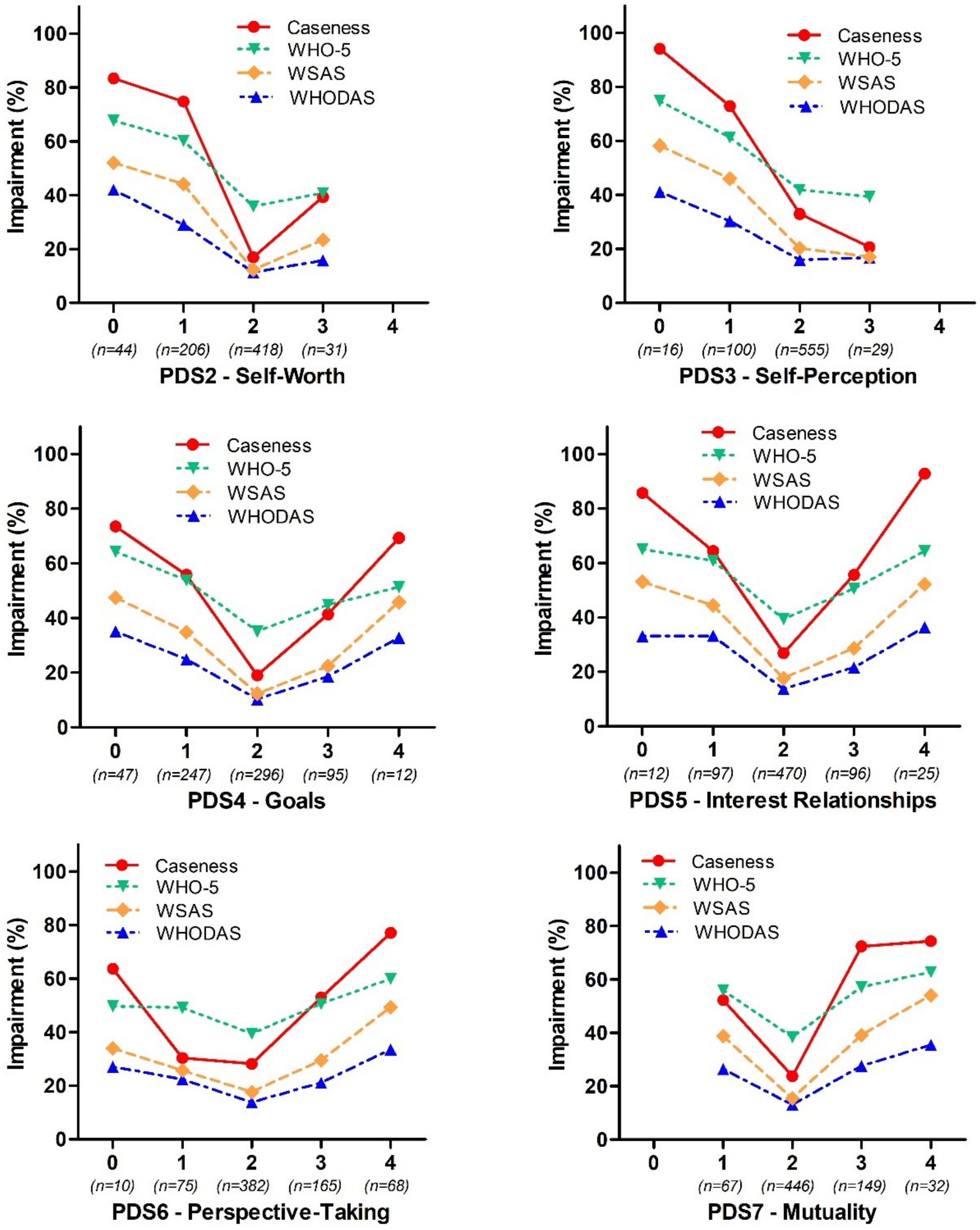

Supplementary Figure S4 ***(cont).***
Level of impairment (%) for the 14 items of the PDS-ICD-11.

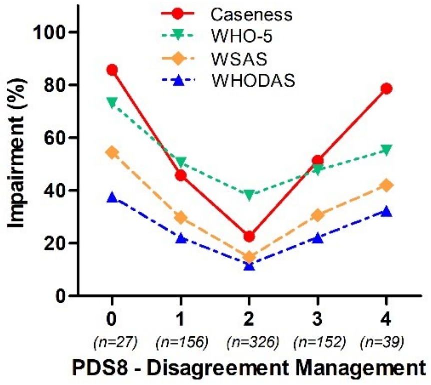

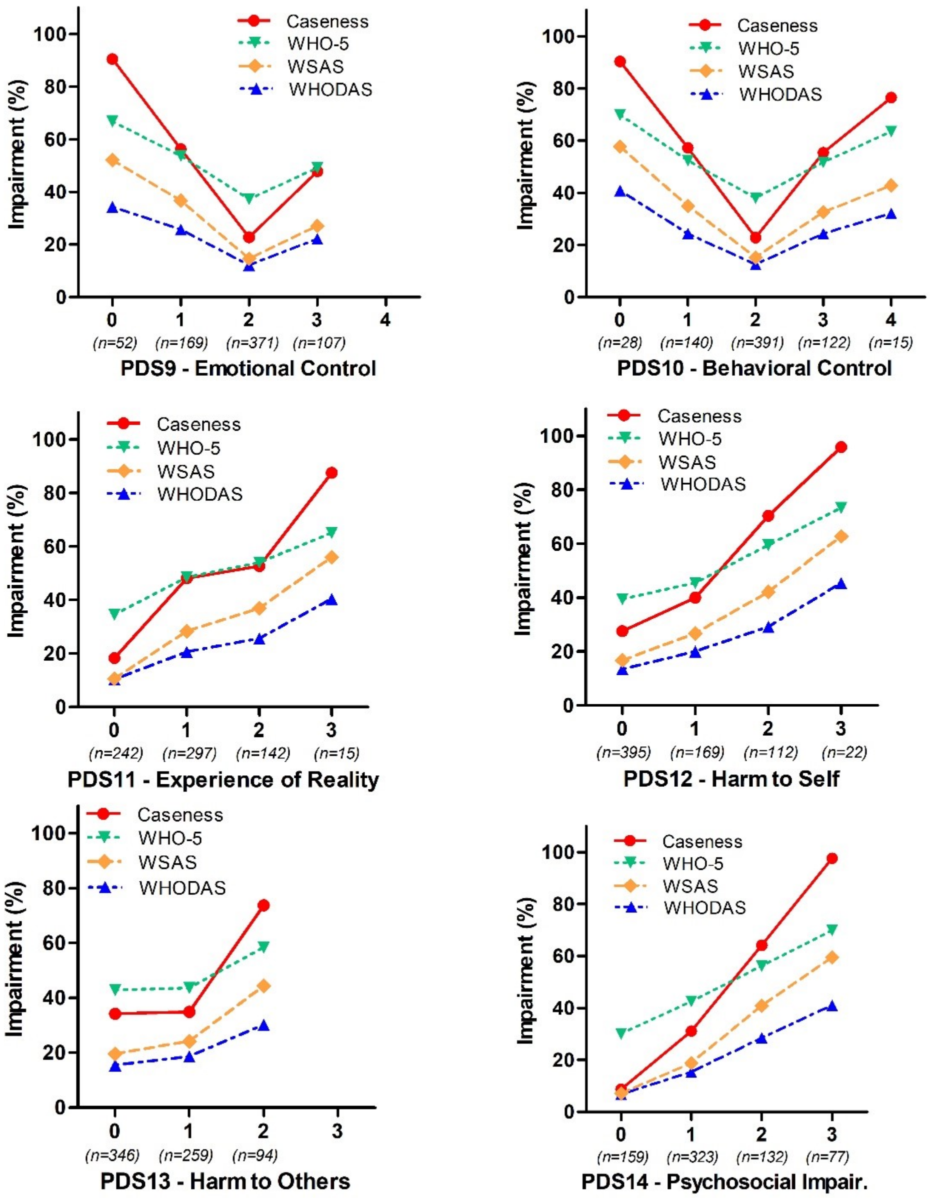

Note. All disfunction scales have been transformed to percentages. WHO-5 scale has been inverted. Categories with n<10 have been collapsed with the nearest category.
